# Engineering a Human Pluripotent Stem Cell-Based *in vitro* Microphysiological System for Studying the Metformin Response in Aortic Smooth Muscle Cells

**DOI:** 10.3389/fbioe.2021.627877

**Published:** 2021-03-18

**Authors:** Nan Chen, Mieradilijiang Abudupataer, Sisi Feng, Shichao Zhu, Wenrui Ma, Jun Li, Hao Lai, Kai Zhu, Chunsheng Wang

**Affiliations:** ^1^Department of Cardiac Surgery, Zhongshan Hospital, Fudan University, Shanghai, China; ^2^Shanghai Institute of Cardiovascular Diseases, Shanghai, China; ^3^State Key Laboratory of Cell Biology, CAS Center for Excellence in Molecular Cell Science, Institute of Biochemistry and Cell Biology, University of Chinese Academy of Sciences, Chinese Academy of Sciences, Shanghai, China

**Keywords:** aortic aneurysm, metformin, drug screening, human pluripotent stem cells, microphysiological system

## Abstract

Aortic aneurysm is a common cardiovascular disease characterised by continuous dilation of the aorta, and this disease places a heavy burden on healthcare worldwide. Few drugs have been suggested to be effective in controlling the progression of aortic aneurysms. Preclinical drug responses from traditional cell culture and animals are usually controversial. An effective *in vitro* model is of great demand for successful drug screening. In this study, we induced an *in vitro* microphysiological system to test metformin, which is a potential drug for the treatment of aortic aneurysms. Human pluripotent stem cell-derived aortic smooth muscle cells (hPSC-HASMCs) were cultured on an *in vitro* microphysiological system, which could replicate the cyclic stretch of the human native aortic wall. By using this system, we found that HASMCs were more likely to present a physiologically contractile phenotype compared to static cell cultures. Moreover, we used hPSC-HASMCs in our microphysiological system to perform metformin drug screening. The results showed that hPSC-HASMCs presented a more contractile phenotype via NOTCH 1 signalling while being treated with metformin. This result indicated that metformin could be utilised to rescue hPSC-HASMCs from phenotype switching during aortic aneurysm progression. This study helps to elucidate potential drug targets for the treatment of aortic aneurysms.

## Introduction

Aortic aneurysm, which is defined as a pathological dilation of the aorta, presents as a life-threatening disease due to the potential to develop dissection or rupture (Goldfinger et al., 2014). Recent studies suggest an average annual death rate increasing by 12% to 2.8/100000 in the last two decades due to aortic aneurysm (Sampson et al., 2014). However, clinical management that has been proven to be curative of aortic aneurysms is limited to surgical replacement or endovascular repair. Thus, great interest in discovering drug therapies that may be positive for reducing continuous dilatation of aortic aneurysms has been shown in many studies (Sweeting et al., 2012). Few efficiencies in reducing aortic aneurysm growth or rupture were associated with these prescribed drugs, including angiotensin-converting enzyme inhibitors, β-blockers, calcium channel blockers and antiplatelet agents (Golledge, 2019). Recently, some clinical studies focusing on the reduction of aortic aneurysm growth in patients receiving metformin prescription were reported (Fujimura et al., 2016; Golledge et al., 2017; Hinchliffe, 2017; Yu et al., 2019). In contrast, an epidemiological study reported that a non-significantly reduced risk of aortic aneurysm rupture was associated with metformin prescription (Kristensen et al., 2017). To address whether the prolonged progress of aortic aneurysm is associated with metformin prescription, a number of randomised, controlled trials (RCTs) are being developed to date.

Some emerging biotechnologies associated with drug screening *in vitro* have been developed. Benefitting from the development of microfluidics, the combination of these techniques can mimic the biological environment of an organ or multiple organs in an *in vitro* microphysiological system, which can be utilised in understanding disease mechanisms and drug effects *in vivo* (Sackmann et al., 2014). Different from traditional *in vitro* models, microphysiological systems can replicate the biomechanical parameters of the human body, which cannot be achieved in 2D cell culture (Horvath et al., 2016; Fang and Eglen, 2017; Xiao et al., 2017). Moreover, microphysiological systems are more cost-effective than *in vivo* experiments, including animal models and preclinical trials in humans. Thus, these emerging techniques can significantly improve the testing efficacy compared to traditional biotechniques. To date, several reports have established different kinds of microphysiological *in vitro* models and related disease models (Huh et al., 2010; Yoon No et al., 2015; Jastrzebska et al., 2016; Musah et al., 2017; Bein et al., 2018). For instance, a vascular micro-physiological system was applied in the construction of models simulating vascular biology, including a biological pulse, cyclic stretching and even shear stress, achieving relevant biophysical conditions for cardiovascular study with cheaper, smaller sample volumes and more precise control compared with animal models and preclinical trails (Beebe et al., 2002; Plouffe et al., 2009).

**FIGURE 1:**
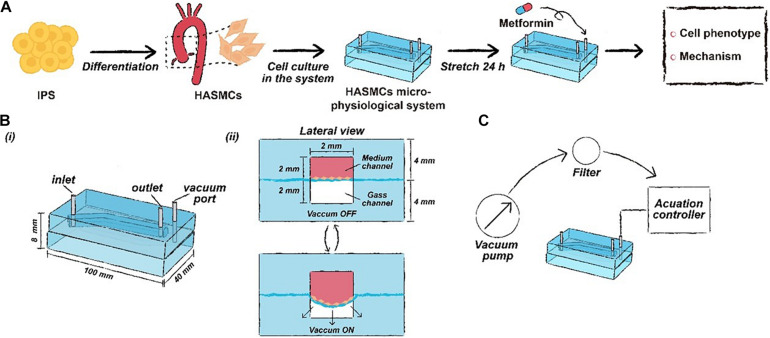
The schematic workflow of this study. **(A)** Drug functional testing of metformin performed in the hPSC-HASMC microphysiological system. **(B)** The parameters of the microphysiological system. **(C)** The schematic of the microfluidic based microphysiological system.

Based on this evidence, we attempted to detect the drug effect of metformin prescription on the progression of aortic aneurysms using a microphysiological system. In this study, we established a microphysiological *in vitro* model associated with the microfluidic technique. The simulation of the *in vivo* pulse and cyclic stretch function of this *in vitro* model was evaluated with human aortic smooth muscle cells (HASMCs). Furthermore, we used human pluripotent stem cells (hPSCs) to generate HASMCs according to a published protocol to detect the drug effect of metformin in the established system. As a result, the hPSC-HASMCs were switched to a contractile phenotype associated with cyclic stretching and metformin prescription. Moreover, we found that the phenotypic switch may be related to the presence of NOTCH 1 signalling, showing the potential to serve as a therapeutic target in preventing the progression of aortic aneurysm. The schematic of our study is shown in Figure 1.

## Materials and Methods

### Design and Fabrication of the HASMC Microphysiological System

The framework of the microphysiological system (100 × 40 × 8 mm) was polydimethylsiloxane (PDMS) carved from polymethyl methacrylate (PMMA) moulds. The PMMA moulds were engraved utilising a computer numerical control (CNC) machine (Jingyan Technology). Then, the PDMS was cast at a ratio of 10:1 (w/w) monomer to curing agent in PMMA moulds and crosslinked at 70°C for 2 h. There were two layers of PDMS in the microdevice, with the top layer containing a medium channel (70 × 2 × 2 mm) and the bottom layer containing a gas channel (70 × 2 × 2 mm). After the construction of the two layers, the bottom layer was attached to the commercialised PDMS membrane using oxygen plasma (Harrick Plasma). Then, the top layer was attached to the bottom membrane layer with oxygen plasma. Finally, the medium channel and the gas channel were connected to different pumps, providing a medium flow and cyclic negative pressure.

### Cell Culture of HASMCs

The HASMC cell line (CRL1999) was purchased from the American Type Culture Collection (ATCC). Smooth muscle cell medium (SMCM, ScienCell) supplemented with 10% (FBS) was used to maintain cell proliferation. The medium channel was coated with collagen (80 μg/mL) and subsequently incubated for 1 h. Then, the microdevice was dried at 70°C for 2 h, and the medium channel was washed with phosphate-buffered saline (PBS). HASMCs were seeded at a density of 2 × 10^6^ per mL in the medium channel. After seeding, the microdevice was incubated at 37°C and 5% CO_2_ for 24 h. Finally, the microdevice system was ready for mechanical stimulation experiments.

### Human PSC Maintenance and hPSC-HASMC Differentiation

The human embryonic stem cell line H9 was provided by the Core Facility for Stem Cell Research (Shanghai Institute of Biochemistry and Cell Biology, China) and selected for differentiation. hPSCs were cultured in mTeSR1 media (StemCell Technologies), passaged with Accutase (StemCell Technologies) and seeded at a density of 3,7000 cells per square centimetre. For differentiation, hPSCs were dissociated with Accutase and plated on Matrigel (BD Biosciences)-coated plates at a density of 3,7000 cells per square centimetre in mTeSR1 media supplied with Y-27632 (10 mM, Selleck). The next day, the medium was refreshed with Mesoderm Specification Medium, which was a mixture of DMEM/F12 and neurobasal media (1:1) supplemented with 2 mM Glutamax, 1× N2 (Life Technologies), 1× B27 (Life Technologies), CHIR99021 (8 mM, Selleck) and BMP4 (25 ng/ml, R&D Systems). After 3 days, the Mesoderm Specification Medium was changed to Vessel Smooth Muscle Cells (VSMCs) Induction Medium, which was a mixture of DMEM/F12 and neurobasal media (1:1) with 1× N2 (Life Technologies), 1× B27 (Life Technologies), PDGF-BB (10 ng/ml, Pepro Tech) and Activin A (2 ng/ml, Pepro Tech). The medium was refreshed every other day. Until day 6, cultures were passaged with Accutase and reseeded at 35,000 cells per square centimetre on gelatine-coated 6-well plates in Vessel Smooth Muscle Cells (VSMCs) Induction Medium reduced Activin A (2 ng/ml, Pepro Tech) to obtain synthetic hPSC-HASMCs. After the hPSC-HASMCs were obtained, the seeding and culturing of the cells in the microdevice system was the same as that of the HASMCs cell line.

### Simulation of Vessel-Like Cyclic Stretching

The simulation of cyclic stretching of the cells was achieved by applying cyclic strain derived from the negative pressure in the gas channel using a microfluidic pump. The pump was connected to the computer-controlled solenoid system, which can provide cyclic stretching with various frequencies (0.5, 1 and 2 Hz). We used a pressure regulator to adjust the negative pressure. Static conditions (0% strain, i.e., 0 kPa, meaning *in vitro* experiments) and cyclic stretch conditions (15% strain, i.e., approximately 6.75 KPa, meaning *in vivo* experiments) were used throughout this study. The normal deformation of the native aorta was reported to range from 9 to 16% in previous studies (Stefanadis et al., 1995; Williams, 1998). We measured the deformation of the PDMS membrane on the cross section of the microphysiological system and the corresponding negative pressure. Then, the relationship between them was characterised. We found that when the deformation of the PDMS membrane was 15%, the corresponding vacuum pressure was 6.75 kPa (Supplementary Figure S1). Thus, we set 6.75 kPa, which can be treated as a theoretically normal strain, as the negative pressure in the stretching group. After 24 h of cyclic stretching, samples were collected for immunofluorescence, RT-qPCR and western blotting analyses.

### Knockdown of NOTCH 1 in hPSC-HASMCs

NOTCH 1-targeted short hairpin RNAs (shRNA, hU6-MCS-Ubiquitin-EGFP-IRES-puromycin) were designed and composed by GeneChem (Shanghai, China). hPSC-HASMCs were seeded into 6-well plates (6 × 10^5^ cells per well) and incubated in a cell incubator. Cells were divided into 3 groups, including the control group, shRNA control (scrambled) group and NOTCH 1 knockdown group (NOTCH 1-KD), when they reached 30% confluency. The shRNA control and NOTCH 1-KD groups were infected with LV-non-specific shRNA and LV-shRNA-NOTCH-1 at an MOI of 10 according to the product manual. After an infection time of 12 h, virus particles were removed from the respective wells, and then, the wells were replenished with fresh SMCM. Cells were further cultured for 72 h in cell culture incubation. Then, the cells were treated with 2.0 μg/mL puromycin, and GFP-positive cells were selected. After the GFP-positive cells reached approximately 80% confluency, they were harvested. The efficiency of NOTCH 1 knockdown was verified by RT-qPCR and western blotting analyses.

### Drug Screening

Metformin (Selleck) was dissolved in deionised water to an initial concentration of 50 mM and stored at −20°C after allocation. We determined the optimal concentration of metformin through RT-qPCR. Among the concentrations, 2 mM increased the expression of SM22 and CNN1 maximally (Supplementary Figure S2). A 2 mM final concentration of metformin was made with fresh medium before use. When the attachment of cells to the PDMS membrane after seeding was achieved (almost 24 h), the medium was exchanged with fresh medium containing metformin at a final concentration (2 mM). Then, cyclic stretching was initiated and lasted for 24 h. The samples were collected for relative analysis after the completion of stretching.

### Cell Viability

After seeding the cells into the microdevice, the viability was analysed at day 1, day 2 and day 3 by a LIVE/DEAD kit (Thermo Fisher Scientific). In brief, the medium channel was gently washed three times with PBS. Second, the microdevice was incubated in the dark for 30 min with the working solution soaking the medium channel. Then, the medium channel was washed three times with PBS to remove the remaining reagents. Finally, the microdevice was disassembled for better observation of the viability in the microdevice using a fluorescence microscope (Leica, DMi8). ImageJ software was utilised to analyse the results.

### Immunofluorescence Analysis

Immunofluorescence analysis was performed directly with a microdevice. The medium was removed, and the medium channel was washed with PBS. The cells were fixed with 4% paraformaldehyde (Sigma-Aldrich) for 30 min at 37°C. Then, the cells were treated with Triton X-100 (1%, Sigma-Aldrich) for 15 min. Bovine serum albumin (5%, Sigma-Aldrich) was selected as the blocking solution and used to treat the cells for 30 min at room temperature. Incubation overnight at 4°C with primary antibodies was performed following blocking. The microdevices were washed three times with PBS and treated with secondary antibodies (1:300) (Alexa 488 anti-rabbit, Thermo Fisher Scientific) for 1 h at room temperature. Nuclei were dyed with 4,6-diamidino-2-phenyllindole (DAPI, Thermo Fisher Scientific) for 15 min. Rhodamine phalloidin (F-actin, Thermo Fisher Scientific) staining was performed to identify the cytoskeletons of the analysed cells. The microdevice was disassembled when the staining was completed. The disassembled microdevice can be more easily observed with a fluorescence microscope (Leica DMi8). ImageJ software was utilised to analyse the obtained images.

### RT-qPCR

RNA was collected from the microdevice by TRIzol (Invitrogen) after 24 h of cyclic stretching. Complementary DNA (cDNA) was synthesised with a PrimeScript RT reagent kit (Takara) according to the product manual. Real-time PCR was performed with TB Green Premix Ex Taq. RT-PCR assays were repeated for triplicate samples of each target gene. Gene expression was normalised using housekeeping glyceraldehyde 3-phosphate dehydrogenase (GAPDH). All primer sequences for the analysed genes are listed in Supplementary Table S1.

### Western Blot Analyses

Cells were lysed by ultrasonication on ice with RIPA (Beyotime, Shanghai, China) lysis buffer supplemented with phenylmethylsulfonyl fluoride (PMSF) protease inhibitor. To obtain an appropriate concentration of protein for western blotting, we lysed cells from 3 microdevices together. A follow-up incubation for 30 min on ice was performed for complete lysis of the cells. The extracts were harvested at a speed of 14,000 rpm at 4°C for 20 min, and the supernatants were collected. Then, the supernatants were treated with a BCA Protein Assay kit (Thermo Fisher Scientific) to quantify the protein concentrations using a spectrophotometric plate reader. The obtained proteins were diluted with loading buffer and heated for 5 min at 95°C. Then, 10 μg of each protein sample was separated by 10% SDS-PAGE and subsequently transferred to 0.2-μm polyvinylidene fluoride (PVDF) membranes (Millipore, Sigma-Aldrich). The PVDF membranes were blocked with non-fat dry milk (5%) for 1 h at room temperature and then incubated with different primary antibodies (Supplementary Table S2) overnight at 4°C. Then, the membranes were incubated with horseradish peroxidase-conjugated goat anti-rabbit (1:6000) and goat anti-mouse IgG secondary antibodies (Cell Signaling Technology) for 1 h at room temperature. The membranes were treated with Super Signal chemiluminescence reagent substrate (Millipore). The protein concentration was normalised using housekeeping β-actin protein. Quantitative evaluation of the intensity of the signals was performed with ImageJ software.

### Statistical Analyses

The results were shown as the mean ± SD. All the experiments in our study were repeated twice, and at least three biological replicates were used in each experiment. Statistical analyses were performed with GraphPad Prism 8 software. Two-tailed Student’s *t* tests were used to evaluate the difference between two groups, and one-way ANOVA followed by Tukey’s *post hoc* tests was used to evaluate the difference between more than two groups.

## Results

### Construction of HASMCs Microphysiological System

**FIGURE 2:**
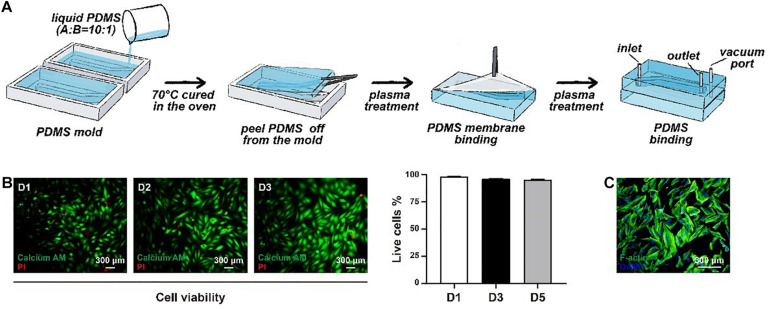
The construction of the microphysiological system. **(A)** The framework of the system was fabricated using elastic PDMS. **(B)** The viability of HASMCs was detected after seeding on days 1, 2 and 3. **(C)** F-actin and DAPI staining of HASMCs in the microphysiological system on day 3.

It is known that the aorta undergoes periodic contraction and relaxation at the same frequency as the cardiac cycle. As the main component in the middle layer of the aorta, HASMCs are also stimulated by cyclic stretching *in vivo*, which cannot be simulated under traditional 2D cell culture. Therefore, we used flexible PDMS to fabricate a two-layer structure of the framework in a microphysiological system to mimic cyclic beating conditions (Figure 2A). There was an elastic PDMS membrane in the middle of the top and bottom layers, dividing the inside chamber into medium and gas channels. The gas channel was controlled with a computer-controlled solenoid system. A change in pressure in the gas channel led to the deformation of the elastic PDMS membrane, which could mimic contraction and relaxation of the aorta *in vivo*. The parameters of the microdevice design are shown in Figure 1B, and the fabrication of the microphysiological system is described in detail in Figure 2A. For the medium channel, we used a peristaltic pump to control the flow. We first used HASMCs (CRL1999) to evaluate the system. Cells showed high viability (≥90%) in the microdevice system at days 1, 2 and 3 (Figure 2B). Furthermore, we stained the HASMCs with F-actin and DAPI, showing the good morphology of the seeded HASMCs (Figure 2C).

### Effect of Cyclic Strain on HASMCs

**FIGURE 3:**
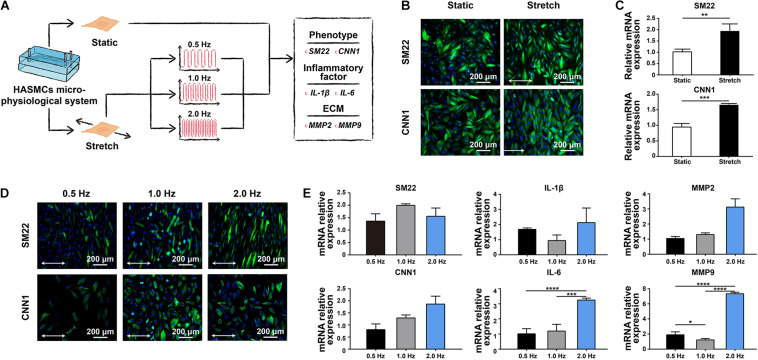
The effect of cyclic stretching on the biology of HASMCs. **(A)** Schematic workflow showing the detection of the effect of cyclic stretching on HASMCs. **(B)** Immunofluorescence staining of SM22 and CNN1 in both the static and stretching culture groups. **(C)** The mRNA expression of SM22 and CNN1 analysed by RT-qPCR. **(D)** Immunofluorescence staining of SM22 and CNN1 in groups treated with different stretching frequencies. **(E)** The mRNA expression of SM22, CNN1, IL-1b, IL-6, MMP-2 and MMP-9 analysed by RT-qPCR. *P < 0.05, **P < 0.01, ***P < 0.001 and ****P < 0.0001.

There are two phenotypes, contractile and synthetic, interact with different environmental simulations in HASMCs. The transition between these two states is called a phenotypic switch, which is essential for HASMCs to perform normal biofunctions *in vivo*. To evaluate the effect of cyclic strain on HASMCs, we first detected the influence of stretching treatment with a fixed frequency at 1 Hz on the cells (Figure 3A). HASMCs were divided into two groups: the static group and the stretching group. The phenotypic switch was evidenced by immunofluorescence staining and the expression of SM22 and CNN1 (contraction markers in HASMCs). As a result, the number of SM22- and CNN1-positive cells in the stretching group (stretching for 24 h) increased significantly compared to that in the static group (Figure 3B and Supplementary Figures S4A,B). The expression levels of SM22 and CNN1 presented a remarkable increase in the stretching group, which is consistent with the results of immunofluorescence staining (Figure 3C). Moreover, we tested the influence of different stretching frequencies (0.5, 1 and 2 Hz) with a fixed stretch (15%, stretching for 24 h) on the cells. The results of immunofluorescence staining showed that there were more SM22-positive HASMCs in the 1 Hz and 2 Hz groups than in the 0.5 Hz groups, while no significant difference was found between these groups (Figure 3D and Supplementary Figure S4C). In terms of the expression of SM22, the results of RT-qPCR showed a similar trend (Figure 3E). In addition, the results of immunofluorescence staining showed that there were more CNN1-positive HASMCs in the 1 Hz and 2 Hz groups than in the 0.5 Hz groups, while no significant difference was found between these groups (Figure 3D and Supplementary Figure S4D). In terms of the expression of CNN1, the results of RT-qPCR showed that higher expression was accompanied with higher stretching frequencies, while no significant difference was found (Figure 3E). To determine an appropriate frequency of cyclic stretching in our study, we subsequently explored the expression of IL-1β, IL-6, MMP-2 and MMP-9 in different groups. As the stretching frequency increased, the expression of IL-6 (an inflammatory marker) increased significantly (Figure 3E). Furthermore, the expression of the extracellular matrix (ECM)-related proteins MMP-2 and MMP-9 showed a remarkable increase with a higher frequency of cyclic stretching (Figure 3E).

### Efficient Differentiation of hPSCs Into HASMCs

To differentiate different hPSCs, a 2D differentiation system was developed to generate mesoderm and LM-HASMCs (Supplementary Figure S2A). First, hPSCs were plated as single cells at a density of 3,7000 cells per square centimetre. Then, cells were differentiated into mesoderm under CHIR and BMP4 treatment. Finally, after PDGF-BB and Activin A induction, mesoderm cells gradually turned into hPSC-HASMCs (Supplementary Figure S2B). During differentiation, mesoderm specification was characterised by dramatic upregulation of the mesodermal marker NK2.5 at day 3, and vascular smooth muscle formation was identified by the upregulation of VSMCs-associated genes, such as SM22, ACAT2 and CNN1 (Supplementary Figure S2C). Immunostaining of hPSC-HASMCs on day 5 showed high levels of calponin, Nestin and SM22 expression (Supplementary Figure S2D). Furthermore, flow cytometry analyses revealed that approximately 99% of cells were positive for a-SMA, which is a marker of vascular smooth muscle (Supplementary Figure S3E). Thus, we were able to generate hPSC-HASMCs from hPSCs with high purity.

### Metformin Switched hPSC-HASMCs to a Contractile Phenotype via NOTCH 1

**FIGURE 4:**
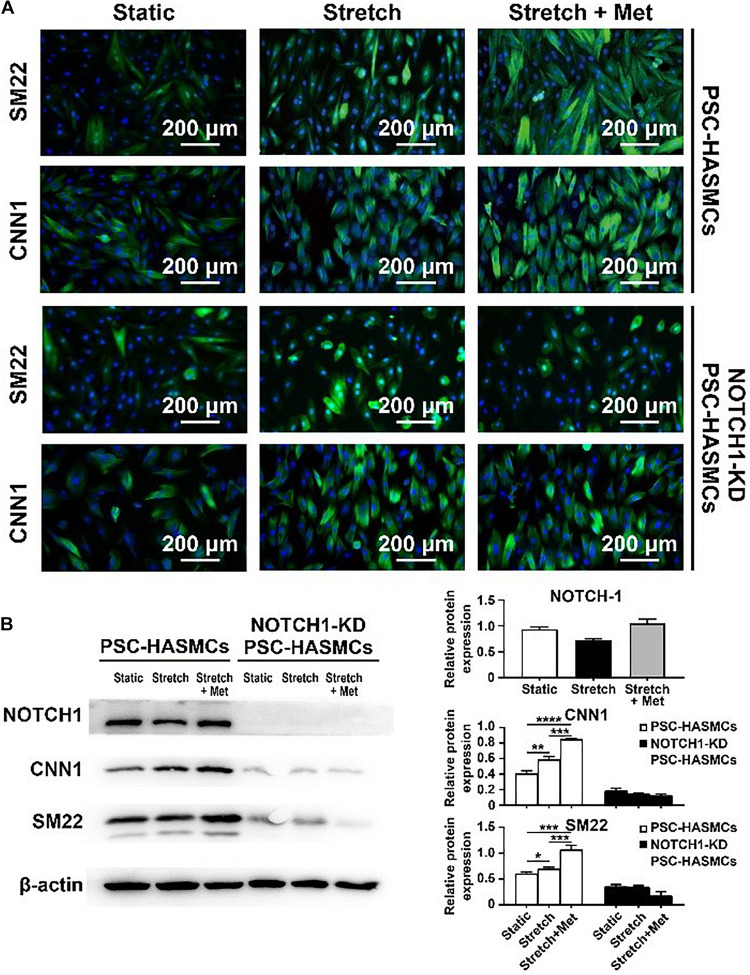
Drug functional testing of metformin in the hPSC-HASMC microphysiological system. **(A)** Immunofluorescence staining of SM22 and CNN1 in hPSC-HASMCs or NOTCH 1-KD hPSC-HASMCs under static, stretching or stretching with metformin treatment. **(B)** The western blot results and statistical analyses of western blots of NOTCH 1, SM22 and CNN1 in different groups. *P < 0.05, **P < 0.01, ***P < 0.001 and ****P < 0.0001.

The main objective of this study was to detect whether metformin prescription could retard the progression of aortic aneurysm and discover the associated mechanism. We seeded the obtained hPSC-HASMCs into the assembled microdevice and connected it to a microfluidic system. Three groups (static group, stretch group and stretch + Met group) were established to evaluate the protective effect of metformin on hPSC-HASMCs. The static group was treated with nothing. The stretch group was treated with cyclic stretching. The stretch + Met group was treated with cyclic stretching and additional metformin. The stretching frequency was set at 1 Hz, and the period of stretching was 24 h. The results of immunofluorescence staining (Figure 4A) and subsequent western blots (Figure 4B) showed that the number of SM22- and CNN1-positive cells increased accordingly with stretching and metformin treatment. This result indicated that cyclic stretching and additional metformin treatment could switch more hPSC-HASMCs to a contractile phenotype. To detect the underlying mechanism, we performed a NOTCH 1 knockdown assay in hPSC-HASMCs (NOTCH 1-KD-hPSC-HASMCs). The same treatments were performed in NOTCH 1-KD-hPSC-HASMCs, as mentioned above. The results of immunofluorescence staining showed that the additional metformin treatment could not induce the phenotypic switch of the hPSC-HASMCs with the knockdown of NOTCH 1 (Figure 4A). Furthermore, the western blot analysis also presented no relevant level-up of the contractile proteins SM22 and CNN1 in NOTCH 1-KD-hPSC-HASMCs with additional metformin treatment (Figure 4B). Based on this evidence, we deduced that metformin may protect hPSC-HASMCs by switching them to a contractile phenotype via NOTCH 1 to delay the progression of aortic aneurysm.

## Discussion

HASMCs in native aortae usually present with a contractile phenotype that allows the vessel wall to withstand cyclic strain from blood pressure (Owens et al., 2004). It has been reported that HASMCs would switch to a synthetic phenotype under pathological conditions. The phenotypic switch from contractile to synthetic will subsequently initiate aneurysms in the aorta under pathological conditions (Petsophonsakul et al., 2019). The results of our study showed that stretching treatment could switch more HASMCs into a contractile phenotype, which is closer to native conditions than those achieved in traditional 2D cell culture. Moreover, the stretching frequency also affects the biological behaviour of HASMCs. Under physiological condition, the normal heart rate varied from 60-100 beats per minute. Our research suggested that different stretching frequencies (with the same stretching amplitude) have no significant influence in phenotype switching of HASMCs. While the expression of IL-6 and MMP-9 increased significantly with 2 Hz stretching frequency (that is 120 beats per minute). It indicated that excessive stretching frequency could increase the expression of inflammation which might do harm to the cell biology of HASMCs. Similarly, it has been reported that arrhythmia, such as atrial fibrillation, was accompanied with a higher risk of aortic aneurism (Ramchand et al., 2021). The underlying mechanism maybe the increased inflammation of VSMCs.

Several population screening studies have revealed that diabetes is associated with a reduced incidence of aortic aneurysms and reduced growth of aortic aneurysms. However, some studies have suggested that the reduction in the prevalence and growth of aortic aneurysms in diabetes might result from the treatment rather than the presence of the disease (Golledge, 2019). To the best of our knowledge, among the drug therapies for diabetes, metformin prescription seems to be the most promising candidate associated with limiting the progression of aortic aneurysm. Due to the lack of RCTs with a high level of evidence, there is still a debate on whether metformin prescription could limit the progression of aortic aneurysm. Thus, many studies focusing on drug functional testing of metformin prescriptions are being explored to address this issue. In our study, we decided to perform functional drug testing of metformin with some emerging biotechnologies. Recently, the utilisation of hPSC-HASMCs extracted from patients with Hutchinson-Gilford progeria syndrome (HGPS) was reported to construct a HASMCs based microphysiological system (Ribas et al., 2017). The HGPS microphysiological system was used to explore the influence on vascular pathophysiology induced by gene mutations in HGPS and to detect the effect of cyclic stretching on HASMCs in HGPS. Disease model-based microfluidic chips combined with hPSCs have unique advantages. They can bridge the gap of species differences between mice and humans and overcome the shortcomings of traditional 2D cell culture. On the basis of this evidence, we established an hPCS-HASMC microphysiological system to detect the effects of metformin on the biology of hPSC-HASMCs.

CNN1 and SM22 we used in this study are biomarkers of contractile function in VSMCs and are related to the pathophysiological of blood vessels. In pathological conditions, including hereditary cardiovascular diseases, hypertension, atherosclerosis and other acquired vascular diseases, the expression of contractile proteins (CNN1, SM22, α-SMA, etc.) will decrease, while the expression of synthetic proteins (OPN, MMP-2, MMP-9, etc.) will increase due to genetic defects, abnormal biomechanical environment or inflammation. As a result, VSMCs will subsequently switch to a synthetic phenotype. Thus, the phenotype switch has been used to evaluate the function and pathophysiological of VSMCs according to many studies (Rensen et al., 2007; Anwar et al., 2012; Granata et al., 2017). In our study, we performed immunofluorescence and western blot to detect the expression of CNN1 and SM22 to directly show the contractile function of VSMCs. Moreover, we detected the expression of MMP-2 and MMP-9, which are commonly used as biomarkers of synthetic phenotype of VSMCs, to show the synthetic function of VSMCs as contrary. The results of metformin testing showed that more hPSC-HASMCs switched to a contractile phenotype with additional metformin treatment than with stretching alone. It indicated that metformin prescription might delay the progress of aneurysms by switching more VSMCs to a contractile phenotype.

NOTCH 1 is a transmembrane receptor protein that participates in cell signalling and the control of cell fate. Loss of the NOTCH 1 gene will induce abnormal differentiation of VSMCs, leading to relevant aortic diseases (Alexander and Owens, 2012; van Varik et al., 2012; Koenig et al., 2017). Previous studies have reported a strong pathophysiological association between NOTCH 1 and the biomechanical forces in cells, especially in blood vessels (Tzemos et al., 2008; Harrison et al., 2019). VSMCs are subjected to continuous mechanical force derived from blood flow *in vivo*. Cyclic stretching and shear stress can be induced by endothelial cells and the ECM to VSMCs. It has been established that NOTCH 1 acts as a biomechanical sensor located in the cell membrane. The presence of NOTCH 1 is necessary for initiating biological responses of VSMCs to haemodynamic forces in arteries (Mack et al., 2017). Meanwhile, it has been reported that NOTCH 1 signalling plays a vital role in the regulation of VSMC differentiation and the expression of contractile proteins. Noseda et al. (2006) found that NOTCH 1 activation is needed for expression of the contractile phenotype protein α-SMA in VSMCs via the NOTCH/CSL axis. High et al. (2007) reported that the loss of NOTCH 1 leads to immature differentiation of the induced pluripotent stem cells into SMCs and the decreased expression of α-SMA, SM22 and CNN1. Moreover, several reports found that metformin can affect the proliferation and differentiation of cells through NOTCH signalling. For instance, Chen et al. found that metformin can alter macrophages toward different phenotypes and inhibit the proliferation and migration of HepG2 cells through Notch signalling (Chen et al., 2015). Rana et al. (2020) reported that metformin can activate AMP-Kinase function via Notch signalling to improve angiogenesis in neonatal pulmonary hypertension. On the basis of these evidence, we hypothesised that metformin prescription might delay the progress of aneurysms through upregulating contractile phenotype in VSMCs via NOTCH 1 signalling. In order to verify this hypothesis, we blocked the NOTCH 1 signalling using lentivirus shRNA targeting NOTCH 1 in hPSC-HASMCs to conform whether the effect of metformin would be inhibited. Our results showed that metformin treatment can up-regulate the expression of contractile phenotype in hPSC-HASMCs. While the up-regulation disappeared with the knockdown of NOTCH 1 in hPSC-HASMCs. Thus, we suggested that metformin might delay the progress of aneurysms through enhancing the phenotype switch in VSMCs via NOTCH 1 signalling.

There are still some limitations in our study. Additional detection is needed to investigate a more specific underlying mechanism between metformin treatment and NOTCH 1 signalling. In addition, the phenotypic switch of VSMCs and the relationship between metformin and NOTCH 1 have been observed in our *in vitro* models, and animal models for performing metformin testing are still needed to confirm the therapeutic effects in parallel. Recently, several studies have reported that the response to metformin varies by age in not only mammals but also invertebrates. Elderly individuals are more likely to suffer from aortic aneurysms compared to youths. Detection of the response to metformin in patients with aortic aneurysms at different ages may also make sense in the future.

## Conclusion

We established a hPSC-HASMCs based microphysiological system to perform drug functional testing of metformin in this study. We found that a contractile phenotype of HASMCs was induced by cyclic stretching in our system, which was closer to the native biology of HASMCs than those from traditional 2D cell culture. Moreover, more phenotypic switching into contractile was found in hPSC-HASMCs treated with additional metformin than in hPSC-HASMCs treated with stretching treatment alone. The response of hPSC-HASMCs to metformin was subsequently verified to be related to NOTCH 1 signalling. Thus, as the evidence in our study showed, we inferred that metformin prescription may be able to limit the progress of aortic aneurysm via NOTCH 1 signalling. More importantly, our study provides strong evidence for the development of preclinical biotechnologies, especially for drug screening and pathophysiological research.

## Data Availability Statement

The original contributions presented in the study are included in the article/Supplementary Material, further inquiries can be directed to the corresponding author/s.

## Author Contributions

NC, MA and SF were involved in the completion of the experiments, data analysis and the manuscript preparation. SZ performed the cell culture experiments. WM helped with the data processing. HL and JL helped with the revision of the manuscript. CW and KZ designed the study and contributed to the data analysis and writing of the manuscript. All authors contributed to the article and approved the submitted version.

## Conflict of Interest

The authors declare that the research was conducted in the absence of any commercial or financial relationships that could be construed as a potential conflict of interest.

## Supplementary Material

Supplementary Figure 1 Characterisation of stretching parameters of the microphysiological system.

Supplementary Figure 2 The mRNA expression of SM22 and CNN1 in different concentrations of metformin.

Supplementary Figure 3 The differentiation and characterisation of hPSC-HASMCs.

Supplementary Figure 4 The fluorescence intensity of the images of SM22 and CNN1 in Figure 3.

Supplementary Table 1 Primers used for RT-qPCR.

Supplementary Table 2 Primary antibodies used for western blotting and immunohistochemistry.

Supplementary Video 1 The stretching movement of the microphysiological system.
