# Supplementary material for: Engineering a Human Pluripotent Stem Cell-Based in vitro Microphysiological System for Studying the Metformin Response in Aortic Smooth Muscle Cells
Source: Front Bioeng Biotechnol. 2021 Mar 18;9:627877. doi: 10.3389/fbioe.2021.627877 (PMC8017487; doi:10.3389/fbioe.2021.627877)
Supplement: Supplementary Figure 1 — Characterisation of stretching parameters of the microphysiological system. [file Table_1.DOCX]

Supplementary Material

# Supplementary Figures and Tables

## Supplementary Figures
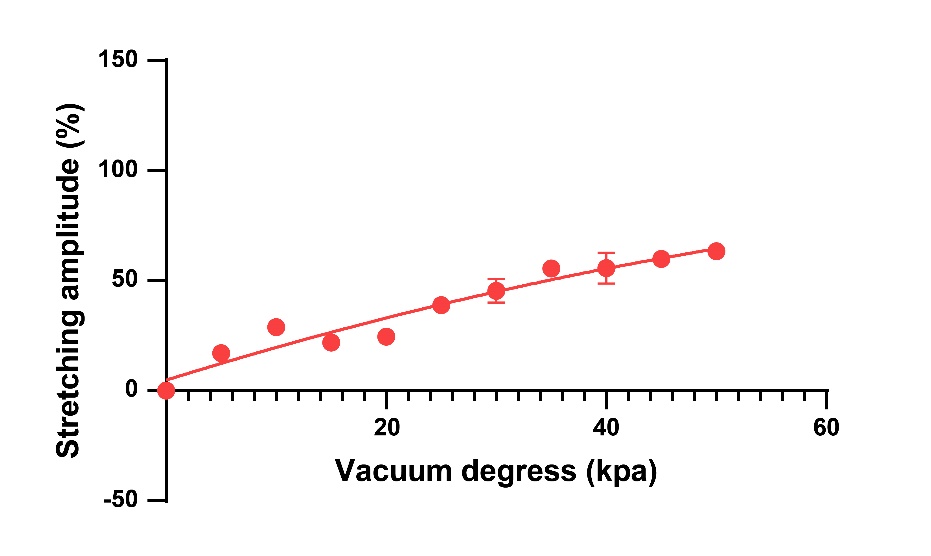


**Figure S1.** **Characterization of stretching parameters of the micro-physiological system.** Membrane deformation under different vacuum pressures (0 kPa, 10 kPa, 15 kPa, 20 kPa).


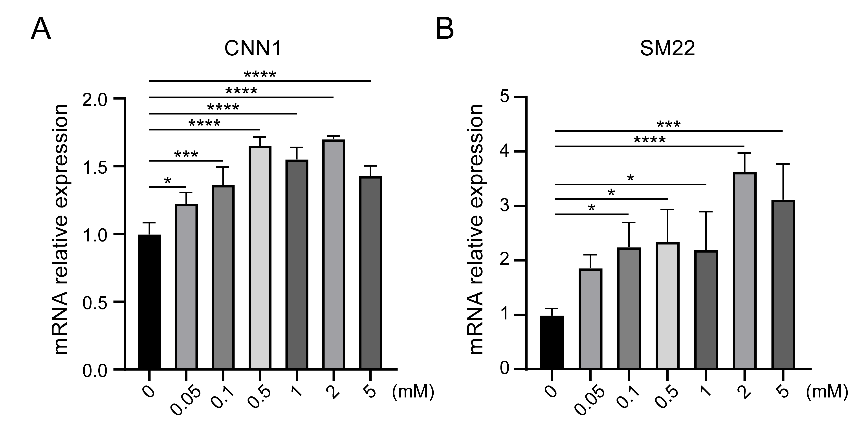


**Figure S2.** **The mRNA expression of SM22 and CNN1 in different concentrations of metformin.** In static condition, the expression of SM22 increased using thses concentrations of metformin treatment, including 0.05, 0.1, 0.5, 1, 2, 5 mM. Among these concentrations, 2 mM increased the expression of SM22 and CNN1 maximally.


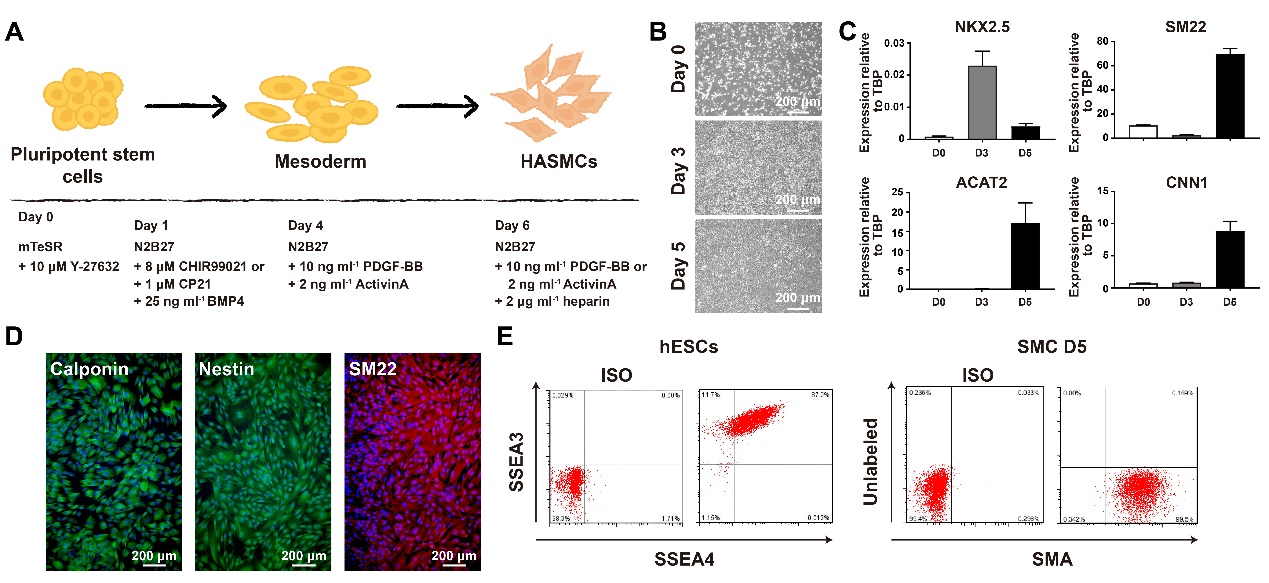


**Figure S3.** **The differentiation and characterization of hPSC-HASMCs.** (A) Schematic of hPSC-HASMCs differentiation system. (B) Morphology of hPSC-HASMCs at day 0, day 3 and day 5. (C) The mRNA expression of SM22, CNN1, ACAT2 and NKX2.5 analyzed by RT-qPCR. (D) The immunofluorescence staining of Nestin, SM22 and CNN1 for hPSC-HASMC. (E) Flow cytometry analyses of vascular smooth muscle marker a-SMA for hPSC-HASMC.


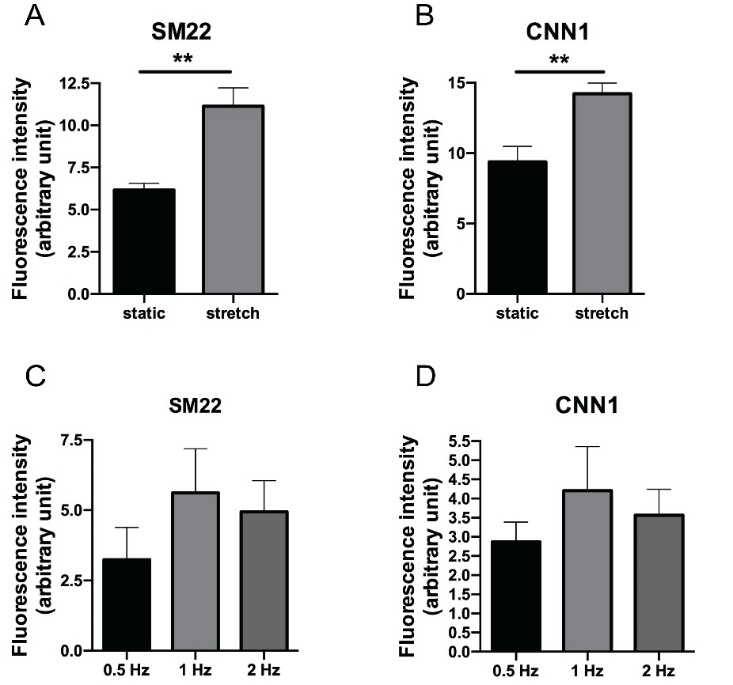


**Figure S4.** **The fluorescence intensity of the images of SM22 and CNN1 in Figure 3.** (A) The immunofluorescence intensity of SM22 in Figure 3B. (B) The immunofluorescence intensity of CNN1 in Figure 3B. (C) The immunofluorescence intensity of SM22 in Figure 3D. (D) The immunofluorescence intensity of CNN1 in Figure 3D. *P<0.05, **P<0.01, ***P<0.001, ****P<0.0001.

**Supplementary Table 1. Primers used for RT-qPCR.**

| **Primers** | **Forward primer** | **Reverse Primer** |
| --- | --- | --- |
| SM22 | CCGTGGAGATCCCAACTGG | CCATCTGAAGGCCAATGACAT |
| CNN1 | CTGTCAGCCGAGGTTAAGAAC | GAGGCCGTCCATGAAGTTGTT |
| IL-1b | AGCTACGAATCTCCGACCAC | CGTTATCCCATGTGTCGAAGAA |
| IL-6 | ACTCACCTCTTCAGAACGAATTG | ACTCACCTCTTCAGAACGAATTG |
| MMP2 | TACAGGATCATTGGCTACACACC | GGTCACATCGCTCCAGACT |
| MMP9 | TGTACCGCTATGGTTACACTCG | TGTACCGCTATGGTTACACTCG |
| GAPDH | CTGGGCTACACTGAGCACC | AAGTGGTCGTTGAGGGCAATG |

**Supplementary Table 2. Primary antibodies used for western blot and immunohistochemistry.**

| **Antibody** | **Company (Cat. No.)** | **Working dilutions** |
| --- | --- | --- |
| NOTCH1 | Abcam (ab52627) | WB:1/1000 |
| SM22 | Abcam (ab14106) | WB:1/1000 IF: 1/300 |
| CNN1 | Abcam (ab46794) | WB:1/1000 IF: 1/300 |
| β-actin | Proteintech (HRP-60008) | WB:1/5000 |


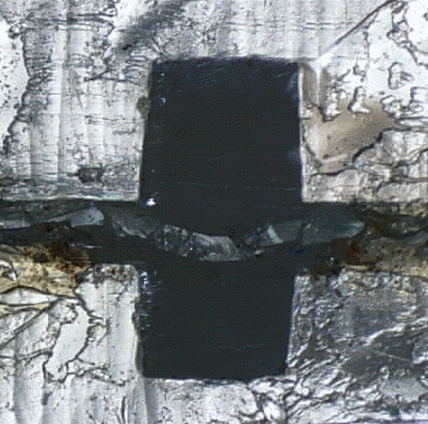


**Video S1. The stretching movement of the microphysiological system.**
